# Job burnout among teachers handling English as a foreign language in China: review and prospects

**DOI:** 10.3389/fpsyg.2023.1202830

**Published:** 2023-07-11

**Authors:** Qiangfu Yu, Xiaofeng Yu

**Affiliations:** Faculty of Humanities and Foreign Languages, Xi’an University of Technology, Xi’an, Shaanxi, China

**Keywords:** EFL, job burnout, teacher burnout, EFL teachers in China, review and prospects

## Abstract

In recent years, job burnout of English as a foreign language (EFL) teachers in China has become prominent in the field of education and psychology, with the related research articles generally on the rise. Using the database of Web of Science (WOS) and the sub-database of Chinese Social Sciences Citation Index (CSSCI) in China National Knowledge Infrastructure (CNKI) database, this paper comprehensively reviews the current situation of research on job burnout of EFL teachers in China between 2020 and 2023, from the aspects of research methods, research focuses and research findings. The literature research results show that on the whole, the research on job burnout of EFL teachers in China is still in its infancy, and that the research level is still relatively low. Based on the systematic reviews of the collected studies, we can conclude that although there is no unanimous conclusion between demographic variables and job burnout severity of EFL teachers in China, we can intervene at both the teacher and school levels to alleviate job burnout of EFL teachers. This review paper analyzes some main problems existing in the current research, for example, lack of theoretical construction and guidance, too much concentration on some research topics, lack of diversified and interdisciplinary research methods, lack of longitudinal research, and potential directions for future research are also discussed in the paper.

## Introduction

Teaching has long been regarded as the most prestigious profession in the world, with various reputations and expectations from all sides. However, teaching is also considered to be one of the most stressful professions in the world (Tsutsumi et al., 2002; Johnson, 2005). Although the research on job burnout has a long history, job burnout is still a research hotspot today, because the causes and countermeasures of job burnout vary in different fields and in different periods. Studies have shown that a significant number of teachers consider leaving their jobs because of problems such as teaching workload, self-efficacy, interpersonal relationships, and the unbalance of pay-to-work (Friedman and Farber, 1992; Clandinin et al., 2015; Greenier et al., 2021). Compared with the traditional work pressure, the development of modern technologies has brought new pressure and challenges to teachers, and long-term pressure and anxiety may lead to teacher burnout (Friedman-Krauss et al., 2014), which significantly influences teachers’ mental and physical health, and in turn, impair their students’ mental health (Buchanan et al., 2013; Fathi et al., 2021) and academic achievements (Jennings and Greenberg, 2009).

EFL teaching in China has been undergoing continuous reform with the change of the times. Take college English teaching as an example, the latest College English Teaching Guidelines has been updated to the 2020 edition after a series of reform measures. Every college English teaching reform in China has focused on solving the problems of students’ EFL learning and the development of EFL teachers. The internationalization and informatization of higher education have put forward new requirements for EFL teachers in China. It will take them a relatively long time to transform the teaching objectives, teaching contents, teaching models, teaching methods, and teaching roles. During this process, EFL teachers in China have to go through the bottleneck period of career development and job burnout period. By analyzing the research on job burnout of EFL teachers in China between 2020 and 2023 in the databases of WOS and CSSCI, this review aims to comprehensively review the current situation of job burnout of EFL teachers in China from the aspects of research methods, research focuses and research findings, explore the major problems existing in the current research and suggest directions for future research.

## Teacher burnout

The concept of job burnout was first proposed by Freudenberger (1974), and he believed that job burnout, a psychological construct, is “the state of physical and emotional depletion resulting from conditions of work” (Freudenberger, 1974). Job burnout is one of the major occupational problems (Hajihasani, 2017) and is unanimously perceived as a psychological syndrome by many scholars. For example, Maslach and Jackson (1981) defined job burnout as a psychological syndrome of emotional exhaustion, depersonalization and reduced personal accomplishments, caused by individuals not being able to effectively cope with the various pressures that continue to occur on the job (Maslach et al., 2001). Similarly, Swider and Zimmerman (2010) described it as a psychological syndrome influencing a person’s work outcomes, clients, family, friends and himself/herself. Furthermore, Useche et al. (2015) suggested that the term is a reaction to chronic exposure to occupational stressors. In addition, Anand and Monika (2017) considered job burnout as a poor mental, physical, and behavioral reaction to work-related stressors.

The term of teacher burnout was first proposed by McGuire, president of the American National Education Association (Bardon, 1983). Since then, scholars began to focus on the field of teacher burnout. Farber (1991) pointed out that teacher burnout is the negative emotional changes that teachers show due to work pressure during long-term teaching experience. According to Dunham and Varma (1998), teacher burnout occurs in a long-term and stressful teaching environment, and due to their failure to effectively deal with the problems caused by bad moods, teachers show a kind of physical and psychological negative reaction, which is manifested in disappearance of interest in work, depletion of teaching enthusiasm, indifference to students, and alienation from colleagues. Fiorilli et al. (2017), taking 149 Italian primary school teachers as participants, found teachers were at risk of highly intense burnout syndrome and that teacher burnout partially mediated the relationship between emotional intensity and satisfaction with social support received. Atashpanjeh et al. (2020) found a moderate negative correlation between teacher burnout (emotional exhaustion and depersonalization) and job satisfaction, based on a mixed-methods study conducted on 103 teachers in Iran.

Teacher burnout is a very important problem in education. Because teacher burnout not only has a negative impact on the body and mind of teachers themselves, but also the learning environment created by teachers will have a negative impact on students’ achievements (Jennings and Greenberg, 2009). Even teacher burnout is one of the main reasons for the early career teacher attrition (Clandinin et al., 2015). It can be seen that teacher burnout has a great negative impact on teachers themselves, students and even the society. Therefore, it is of great significance to find out the influencing factors of teacher burnout and the measures to prevent and alleviate teacher burnout.

## Research design

The aim of this review paper is to investigate how the research status on job burnout of EFL teachers in China has evolved between 2020 and 2023, to identify key theories and major findings, explore problems and gaps existing in the current research, and to propose potential directions for future research. To achieve the aim, this paper uses a mixed-methods approach involving both quantitative research through standard databases of WOS and CSSCI and qualitative analysis through iterative reading.

### Data collection

In this review paper, previous studies on job burnout of EFL teachers in China were searched as follows. Firstly, studies published in international journals were searched in electronic databases of Web of Science (WOS). The author used the following searching parameters to conduct the search for previous studies: TI = (burnout) AND TI = (English teacher OR foreign language teacher OR second language teacher) AND SILOID = (WOS) AND PY = (2000–2023). Secondly, studies published in Chinese journals were searched in China National Knowledge Infrastructure (CNKI) database. The author used the Chinese counterparts of the searching parameters mentioned above and extracted the studies on job burnout of EFL teachers in China in Chinese Social Sciences Citation Index (CSSCI) source journals.

**FIGURE 1:**
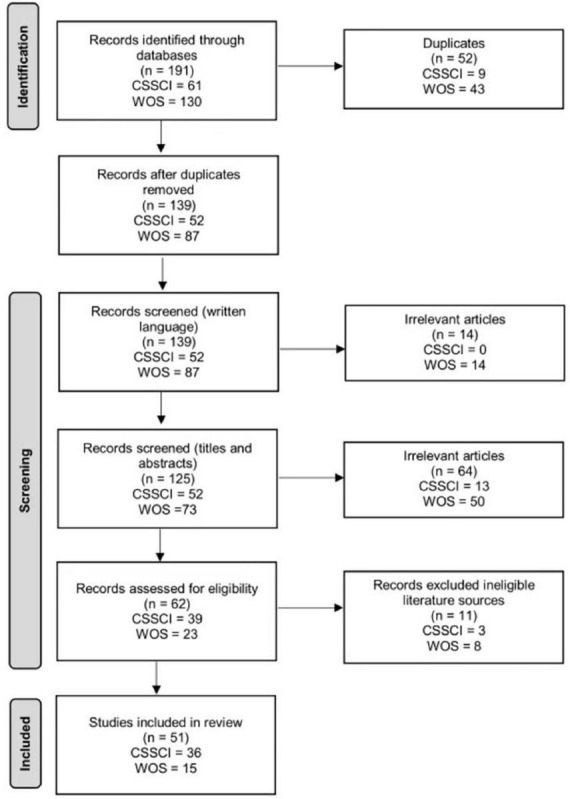
Flow diagram illustrating the process of article selection for review.

Studies were eligible for inclusion if they focused on job burnout of EFL teachers in China and the relevant key words (e.g., China, Chinese) appeared in the titles or abstracts. We included all studies, both speculative or theoretical research design, empirical research design, qualitative and quantitative, in this review. Only CSSCI articles that were published in Chinese and WOS articles published in English were included. Conference proceedings, book reviews and review articles were outside the scope of our study and were therefore excluded from our data collection. Figure 1 summarizes the selection criteria and data collection process (Page et al., 2021).

### Data analysis

To make the review process systematic, transparent and reproducible (Zupic and Èater, 2015) and identify the research streams, this review paper first performed a bibliometric analysis of the studies extracted from the databases of WOS and CSSCI. Then, a systematic coding analysis through iterative reading highlighted the following categories that guided the analysis: (i) speculative or empirical studies; (ii) research methodology; (iii) research focuses; (iv) research findings.

## Results

**FIGURE 2:**
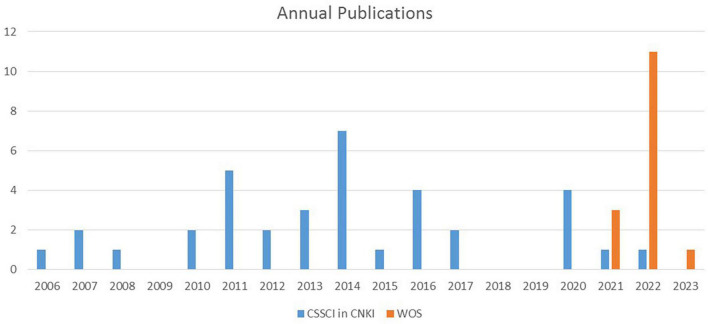
Annual publications for selected journal articles.

As a result, 15 papers were found in WOS, and 36 in CSSCI source journals in CNKI. As is shown in Figure 2, from 2006 to 2010, only 6 articles were published, all of which were published in CSSCI source journals in CNKI; articles published from 2011 to 2017 were on a rise, with the total of 24 published in CSSCI source journals in CNKI; from 2018 to 2023, 21 articles were published, with 6 articles published in CSSCI source journals in CNKI and 15 published in WOS.

Compared with the total of 4,616 articles on teacher burnout and 373 articles on job burnout of EFL teachers in China published in CNKI, only 36 articles on job burnout of EFL teachers in China were published in CSSCI source journals in CNKI and 15 in WOS. It is indicated that on the whole, the research on job burnout of EFL teachers in China is still in its infancy, and the research level is still relatively low. It is necessary to comb and analyze the literature carefully from the aspects of research methods, research focuses and research findings, so as to find the existing problems in the research and explore the enlightenments for future research.

### Research methods of studies on EFL teachers’ burnout in China

Research methods on job burnout of EFL teachers in China have mainly experienced the process from qualitative research to quantitative research, with empirical research method as the mainstream method, which is basically consistent with the overall empirical orientation of research on teacher burnout abroad (Jiang, 2019). And similarly quantitative research has experienced the process from using the scales devised by foreign scholars to using the self-developed scales. Of the 51 selected articles, 13 articles used a speculative or theoretical research design, accounting for 25.49% of the total, and 38 articles used an empirical research design, accounting for 74.51%, with 25 articles (65.79%) using quantitative methods, 5 articles (13.16%) using qualitative methods, and 8 articles (21.05%) using both quantitative and qualitative methods.

#### Speculative studies

Speculative studies on job burnout of EFL teachers in China are mainly focused on definition, causes and countermeasures of teacher burnout. Fan and Yang (2015) defined teacher burnout, arguing that teacher burnout refers to the emotional exhaustion, depersonalization and reduced personal achievements caused by the constant consumption of various pressures and emotional resources arising from work, organization and society, and it is a result of teachers being overwhelmed by work for a long time and failing to cope with a series of emotional, cognitive and behavioral psychological fatigue and tension, thus restricting their professional development. Song (2013) believed that job burnout and professional development are in essence a pair of interrelated categories. Job burnout is not only the biggest obstacle affecting the professional development of college English teachers and college English teaching efficacy, but also the natural result of the weakening of professional development of English teachers. Strengthening teachers’ professional development is an effective way to eliminate teacher burnout (Song, 2013). Based on the influence of job burnout on the professional development of teachers, Hu and Liu (2017) proposed countermeasures to improve the job burnout state of college teachers and promote their professional development, such as creating a reasonable campus mechanism and strengthening teachers’ personal quality.

#### Empirical studies

Empirical studies on job burnout of EFL teachers in China mainly adopt quantitative methods and qualitative methods. As for qualitative studies, a majority of researchers used interviews to analyze the job burnout of EFL teachers in China (Zhang, 2011). A few studies combined interviews with other methods. For example, based on qualitative data including multiple semi-structured interviews, class observations, teacher reflective notes, student feedback, and institutional documents over 18 months, Ding et al. (2022) explored the dynamic and diachronic emotion labor of two veteran English lecturers in Chinese universities and found that the long-term surface acting of depressing negative emotions is positively related to job burnout and negatively correlated to teachers’ efficacy. Wang and Song (2022) adopted a qualitative method of interviews and online classroom observations conducted with 20 Chinese English teachers to explore the changes on EFL teachers’ emotional labor brought about by online teaching, and found that a variety of factors, such as the degree of adaptation to online teaching technology and the invisibility of the online teaching space, contribute to EFL teachers’ emotional labor that can cause job burnout and professional identity dilemmas. Guo (2022) adopted narrative research to narrate the dynamic formation process of a primary school English teacher’s job burnout, through observation, interviews and information analysis of the interviewee’s Wechat, Weibo and Douyin contents.

Since the middle and late 1980s, scholars abroad began to compile and measure job burnout scales, which opened the curtain of quantitative research on teacher burnout. Some influential scales (Pines et al., 1981; Ford et al., 1983; Demerouti et al., 2003; Kristensen et al., 2005) emerged successively, among which the most influential and widely-used one is Maslach Burnout Inventory (MBI) (Maslach and Jackson, 1981, 1986), with its series of scales of MBI-HSS (MBI-Human Services Survey) (Maslach and Jackson, 1996), MBI-ES (MBI-Educators Survey) (Maslach et al., 1996b) and MBI-GS (MBI-General Survey) (Maslach et al., 1996a). Compared with European and American scholars’ research on job burnout scales, Chinese scholars started late, and currently they mainly stay in translation or chinesization (Shao and Gao, 2005; Cheng, 2006; Wu, 2010), revision (Wang et al., 2003, 2005; Zhang et al., 2015), compilation (Li and Wu, 2005; Xu and Han, 2010; Guo and Xu, 2017) of those western scales.

Most quantitative studies on job burnout of EFL teachers in China are based on MBI (Zhang, 2012, 2022; Hu, 2014; Li, 2016; Fan et al., 2017; Li and Zhang, 2020; Bing et al., 2022; Ding and He, 2022; Song, 2022; Yang, 2022). Some empirical studies revised some items of MBT and added some items to highlight China’s national conditions and cultural specificity (Chen and Lin, 2008; Xu, 2010; Yao, 2011; Tang and Zhao, 2013; Tang, 2020). A few scholars independently compiled teacher burnout scales (Wang, 2011; Liu, 2013), but the academic value of the scales still needs further verification.

There are also some studies adopting both qualitative and quantitative methods to explore job burnout of EFL teachers in China, most of which adopted interviews and questionnaire surveys based on MBI (Tang, 2011; Liu L., 2014; Liu P., 2014; Hu, 2016, 2021).

### Research focuses of studies on EFL teachers’ burnout in China

As for research objects of job burnout of EFL teachers in China, 37 articles focused on university EFL teachers’ job burnout, accounting for 72.55% of the total, 5 articles focused on higher vocational college EFL teachers’ job burnout, accounting for 9.8%, 5 articles focused on middle school EFL teachers’ job burnout, accounting for 9.8%, and 4 articles focused on primary school EFL teachers’ job burnout, accounting for 7.84%.

Both the speculative research and the empirical research on job burnout of EFL teachers in China have their own focuses in terms of research contents. The vast majority of speculative research focused either on the causes of job burnout of EFL teachers in China (Wang, 2010; Song, 2013; Jiao, 2014), or on its countermeasures (Zheng and Lou, 2006; Li and Jin, 2014; Fan and Yang, 2015; Jiang, 2016), or on its causes and countermeasures (Li, 2007; Yin, 2011; Zhang, 2014; Bao, 2016; Hu and Liu, 2017; Wang and Wang, 2020).

Most empirical studies focused on the severity, influencing factors and coping strategies of job burnout of EFL teachers in China. Of the 38 empirical research articles, 19 articles analyzed the job burnout severity among EFL teachers in China (Xu, 2010; Tang, 2011; Liu, 2013; Liu P., 2014; Li, 2016; Fan et al., 2017), 29 articles explored the influencing factors of job burnout of EFL teachers in China (Chen and Lin, 2008; Tang and Zhao, 2013; Liu L., 2014, Liu M., 2014; Li and Zhang, 2020), and 27 articles proposed some coping strategies for job burnout of EFL teachers in China (Hu, 2014, 2016, 2021; Tang, 2020).

### Research findings of studies on EFL teachers’ burnout in China

#### Job burnout severity of EFL teachers in China

Generally speaking, EFL teachers in China are in moderate or mild level of job burnout, while the proportion of EFL teachers in high level of job burnout is relatively low. However, from the detection rate of single factor of job burnout, different studies have not reached a consistent conclusion. Tang (2011) evaluated the comprehensive burnout level of 59 college English teachers in 9 universities in China, and found that 69, 46, and 8% of teachers had mild, moderate and high levels of job burnout respectively. And the detection rates of burnout level of reduced personal achievements, emotional exhaustion and depersonalization were 34, 32, and 24% respectively. This is basically consistent with the findings of Xu (2010), who found that the job burnout level of college English teachers was mild to moderate, with a detection rate of 75.4%. However, Chen and Lin (2008) investigated 120 English teachers in 15 higher vocational colleges in Guangzhou and showed that there was obvious job burnout among English teachers in higher vocational colleges. Zhang (2012) investigated 267 college English teachers in Henan Province and also found that the job burnout of college English teachers was at a relatively serious level, with a score of 3.47 in the dimension of emotional exhaustion, 3.11 in the dimension of depersonalization, and 2.87 in the dimension of reduced personal achievements.

However, Li (2016) investigated the job burnout of 155 English teachers in 12 colleges and universities in Guangdong Province, and found that college English teachers have a certain degree of job burnout, and that there are differences in the three dimensions of teacher burnout: emotional exhaustion almost reaches the moderate level, depersonalization reaches the mild level, and reduced personal achievements are at the moderate and mild level. Through the MBI questionnaire survey on 203 English teachers from three colleges and universities in Zhejiang Province, Fan et al. (2017) also found that college English teachers have a certain degree of job burnout, but it is not particularly serious, and the level of emotional exhaustion is also the highest among the three dimensions. However, Hu (2021), based on the questionnaire survey of 297 English major teachers in 18 Chinese public universities and the interviews with 9 of them, found that the overall level of job burnout among English major teachers in universities is not very high. She believed that this may be because teachers with rich teaching experience, relatively low research pressure and senior titles and teachers with teaching experience of more than 15 years account for about one-third of the samples. Moreover, teachers with low professional titles and young teachers still have relatively high enthusiasm and expectation for career development, so the overall job burnout shown in the samples is not serious.

#### Causes of job burnout of EFL teachers in China

Yin (2011) attributed the causes of job burnout of college EFL teachers in China to the increasing requirements of society on college EFL teaching, the challenge of college EFL teaching activities brought by the differences of students’ English proficiency, the improvement of EFL teachers’ teaching assessment standards, the heavy workload of college EFL teachers brought by school enrollment expansion, the emphasis on scientific research over teaching in the job appointment system, the lack of interdisciplinary knowledge structure of college EFL teachers and the individual personality of EFL teachers. Similarly, Bao (2016) believed that job burnout of college EFL teachers can be attributed to the pressure brought by the innovation of college EFL teaching, the impact brought by the adjustment of teaching quality evaluation mechanism, the overload caused by the expansion of colleges and universities, the troubles caused by the evaluation of teachers’ professional titles, and the negative impact brought by the characteristics of teachers’ profession. In addition, Zhang (2011) found that the following pairs of contradictions are the main reasons for the job burnout of college EFL teachers: the contradiction between teachers’ high pay and low return to students, the contradiction between teachers’ repeatability and creativity in teaching work, the contradiction between teachers’ teaching work and their own professional development, the contradiction between teachers’ teaching work and the research-oriented evaluation mechanism, and the contradiction between teachers’ labor remuneration and market demand. Jiao (2014) analyzed the causes of job burnout of EFL teachers from the perspective of ecological philosophy, and found that changes in the niche of EFL teachers as well as the negative energy transmission of information among various factors that constitute the ecosystem of EFL education caused their psychological imbalance, which then led to the generation of their job burnout. Zhang (2014) analyzed the job burnout of young EFL teachers in engineering colleges and universities and its causes from the aspects of students, teachers, schools, society and economic pressure. Wang and Wang (2020) analyzed the causes of occupational burnout of EFL teachers in higher vocational colleges, including low social recognition of higher vocational education, poor quality of students in higher vocational colleges, negative effects brought by curriculum reform and requirements for improving teaching and scientific research ability, imperfect evaluation standards for EFL teachers, and the comprehensive quality of EFL teachers to be improved.

To sum up, job burnout of EFL teachers in China is mainly attributed to weak professional development (such as low educational level, lack of language ability and subject teaching knowledge, lack of training opportunities, low level of scientific research), EFL teachers’ work characteristics (such as heavy teaching workload, conflicts between test-taking pressure and teaching innovation, few opportunities to “recharge”, negative impact of work characteristics on interpersonal relationship, professional title evaluation trouble), and social expectations and pressure (such as the improvement of teaching requirements, the differences of students’ English proficiency affecting the development of teaching activities, and the improvement of EFL teachers’ teaching assessment standards).

#### Factors influencing job burnout of EFL teachers in China

Research on job burnout of EFL teachers in China analyzed the influencing factors mainly though demographic variables, such as gender, age, length of teaching, educational background, professional title, and marital status. However, there is no unanimous conclusion between demographic variables and job burnout severity of EFL teachers in China.

**Gender.** Some studies have shown that there is a significant difference in the level of job burnout between male and female EFL teachers in China. For example, Xu (2010) found that the level of reduced personal accomplishments of female EFL teachers is significantly higher than that of male EFL teachers, while Tang (2011) found that the level of reduced personal accomplishments is significantly lower than that of male counterparts. Li (2016) found that female EFL teachers are more serious than male EFL teachers in terms of emotional exhaustion, and that there is a significant difference between them, but there is no significant difference between them in terms of depersonalization and reduced personal accomplishments. Liu (2013) found that among college EFL teachers, the job burnout level of female teachers was significantly higher than that of male teachers. However, Tang and Zhao (2013) found that male teachers’ job burnout was higher than female teachers, and that male teachers’ average burnout in three dimensions of emotional exhaustion, depersonalization and reduced personal accomplishments was higher than female teachers, and the difference reached a significant degree in the dimension of depersonalization. However, many studies have shown that there is no significant difference in job burnout level between male and female EFL teachers in Chian (Zhang, 2012; Liu L., 2014).

**Age.** Many studies have shown that there are statistically significant differences in teacher burnout levels among different age groups. Liang and Liu (2007) found that among rural middle school teachers in China, the job burnout of 46–50-year-old teachers is the highest, followed by 26–30-year-old teachers, and the job burnout of 31–35-year-old teachers is the lowest. However, Hu (2021) believed that the younger the EFL teachers in colleges and universities are, the lower their educational level and the more serious their depersonalization degree will be. There are also studies showing that the burnout level of EFL teachers in China presents an inverted U-shaped development trend with the growth of age. For example, Tang (2011) found that 30–39-year-old teachers are prone to job burnout, and that their burnout level is the highest among all age groups in terms of emotional exhaustion, depersonalization and reduced personal accomplishments. Yao (2011) believed that EFL teachers aged 30–50 in rural middle schools are prone to job burnout.

**Length of teaching.** Many studies show that there is a significant correlation between length of teaching and job burnout of EFL teachers in China (Ding and Liu, 2020), but the research results on the relationship between length of teaching and job burnout are not the same. Liang and Liu (2007) found that those who taught for 2–5 years and 21–26 years had the highest burnout level, and that the latter was higher than the former. Zhang (2011), Tang and Zhao (2013) also believed that the longer the length of teaching and the richer the teaching experience, the higher the degree of job burnout. However, Liu L. (2014) found that teachers with more than 15 years of teaching experience scored significantly lower than teachers in other age groups in emotional exhaustion, depersonalization and reduced personal accomplishments, and had the lowest level of job burnout. From the perspective of single factor analysis, Xu (2010) showed that there were significant differences in emotional exhaustion of EFL teachers in 5 different age groups, teachers with 16–20 years of teaching experience having significantly higher emotional exhaustion than teachers in other age groups, but there was no significant differences in depersonalization and reduced personal accomplishments. Liu (2013) and Li (2016) came to a similar conclusion in their studies, which respectively concluded that teachers with teaching experience of 15–20 years and more than 16 years had the most severe emotional exhaustion. However, Tang and Zhao (2013) found that the development trend of emotional exhaustion and depersonalization in different teaching age groups basically increased first and then decreased, while the development of reduced personal accomplishments has been continuously strengthened.

**Educational background.** Many studies show that there is a significant negative correlation between job burnout and educational background, and the degree of job burnout among EFL teachers with bachelor’s degree is the highest (Xu, 2010; Liu, 2013; Tang and Zhao, 2013; Hu, 2021). However, the research of Liu L. (2014) showed that college EFL teachers with master’s degree showed the highest degree of overall burnout and the dimension of emotional exhaustion. Ding and Liu (2020) also found that EFL teachers with higher educational background in rural areas of China had significantly higher burnout than those with lower educational background. There are also studies showing that educational background has no significant influence on the burnout level of EFL teachers (Tang, 2011).

**Professional title.** Most studies show that there is no significant correlation between professional title and job burnout of EFL teachers in China (Xu, 2010; Tang, 2011; Liu L., 2014; Ding and Liu, 2020). Other studies have reached different conclusions. For example, Liu (2013) showed that with the promotion of professional titles, EFL teachers are more prominent in emotional exhaustion and depersonalization, while the job burnout of associate professors is at the most serious level. Tang and Zhao (2013) found that teachers with different professional titles have certain differences in the degree of teacher burnout. Lecturers had the highest level of emotional exhaustion, followed by associate professors and teaching assistants. Li (2016) found that teachers with different professional titles had significant differences in emotional exhaustion and reduced personal accomplishments, among which associate professors scored the highest and teaching assistants the lowest.

**Marital status.** A vast majority of studies show that marital status has no significant influence on overall job burnout of EFL teachers in China (Wang, 2011; Tang and Zhao, 2013; Ding and Liu, 2020). From the perspective of single factor analysis, marital status has a significant difference in the dimension of reduced personal accomplishments, and the level of reduced personal accomplishments of married EFL teachers is significantly higher than that of unmarried teachers (Xu, 2010; Wang, 2011).

To sum up, scholars have not reached a consistent conclusion on the correlation research between teacher demographic variables and job burnout of EFL teachers in China, and studies show different linear or non-linear relationships between teacher demographic variables and the three dimensions of job burnout. Although the current research shows that gender has no significant effect on English teachers’ job burnout, many studies have shown that middle-aged and elderly teachers, teachers with more than ten years of teaching experience, teachers with associate professor titles and married teachers are more prone to job burnout.

#### Coping strategies for job burnout of EFL teachers in China

In view of the job burnout of EFL teachers in China, researchers mainly discussed the coping strategies from the two levels of teachers and schools. Firstly, researchers generally agreed that the individual professional development of EFL teachers is the key to alleviating job burnout. Fan and Yang (2015) believed that EFL teachers are the main force for their professional development. Teachers should have lifelong learning and reflective consciousness, pay attention to the development of interpersonal communication and self-management ability, and improve self-efficacy, so as to effectively resist job burnout and realize their own professional development. EFL teachers should also take an active part in training and learning, cultivate the awareness of scientific research (Song, 2013), actively conduct teaching reflection and action research, and adapt to the changes of ecological environment (Li, 2016), so as to reduce the chances of burnout. Liu P. (2014) pointed out that college English teachers should pay attention to cultivating their ability to withstand pressure, enhance their ability to adapt to the environment, respond appropriately and effectively to the reality, and realize the harmony between psychological environment and external environment. Hu and Liu (2017) believed that teachers should start from their own factors, strengthen their personal quality, establish reasonable beliefs and maintain a good physical and mental state.

Secondly, at the school level, the fundamental way to alleviate job burnout of EFL teachers in China lies in the reform of college management system. Many studies have suggested that it is imperative for schools to actively promote the reform of personnel system and improve the measures of performance appraisal management (Bao, 2016; Hu and Liu, 2017; Wang and Wang, 2020; Hu, 2021; Guo, 2022), fully consider the professional characteristics of English teachers, and develop a scientific and reasonable professional title evaluation system (Yin, 2011; Li and Zhang, 2020). In addition, schools should build a professional development platform for EFL teachers and provide them with cognitive and vocational training to protect themselves from job burnout (Fan and Yang, 2015; Li and Zhang, 2020). Universities with qualified conditions should be encouraged to set up foreign language teaching and research journals, so as to provide more teaching and research exchanges for EFL teachers (Yin, 2011). Li and Zhang (2020) believed that schools should earnestly implement the provisions of teacher-student ratio, actively introduce EFL teachers with high education and high level, match the number of EFL teachers, optimize the structure of EFL teachers, and effectively solve the problem of teaching overload of EFL teachers due to insufficient staffing. At the same time, schools should strive to change the classroom environment of EFL teaching in large classes, establish more convenient network and voice teaching platforms, and provide favorable conditions for EFL teachers to carry out new teaching practices such as MOOCs and teaching research and research activities. Ding and Liu (2020) proposed that schools should attach importance to the influence of teacher burnout on students’ achievements, establish a normalized evaluation mechanism for teacher burnout, and commission third-party institutions or school psychological consultation rooms to timely understand the trend of teacher burnout, so as to better prevent and eliminate some burnout emotions of EFL teachers and improve their career happiness.

## Discussions

The research on job burnout of EFL teachers in China has achieved preliminary results. The current level of job burnout of EFL teachers has been gradually clarified, the causes and major factors influencing job burnout of EFL teachers have been basically clarified, and some practical countermeasures have been put forward to prevent or alleviate the job burnout of EFL teachers. However, there are still some problems to be solved in the theoretical guidance, research contents and research methods of job burnout of EFL teachers in China.

First of all, there is still a lack of theoretical guidance and support for the research on job burnout of EFL teachers in China. Foreign empirical studies on teacher burnout attribution have proposed and verified many theoretical models, such as ecological theory of job burnout by Carroll and White (1982), resource conservation theory by Hobfoll (1988, 1989) and social exchange theory by Buunk and Schaufeli (1993). However, of the empirical studies on the attribution of teacher job burnout in China, researchers almost all collected data through interviews or questionnaires, and then conduct data processing and analysis. In fact, researchers can try to build attributional models of EFL teachers’ job burnout under the guidance of relevant theories. The same is true of speculative research. Researchers have not yet explained the deep reasons behind the phenomenon of job burnout of EFL teachers from the theoretical construction. Theoretical construction should be the main direction of speculative research on job burnout of EFL teachers in the future.

Secondly, the topics and contents of researchers are too concentrated. Most of the studies focus on the *status quo* investigation and cause analysis of job burnout of EFL teachers in China. However, most of the studies analyze the causes of job burnout from demography-related variables. To some extent, it is difficult to clarify the common problems of EFL teachers’ job burnout. Moreover, most current studies focus on the job burnout of EFL teachers in colleges and universities, and pay little attention to the job burnout of EFL teachers in primary and secondary schools. Future studies should also increase the study of job burnout of EFL teachers in primary and secondary schools. In addition, there are few studies on the relationship between job burnout and specific influencing factors such as self-efficacy, psychological capital, mental resilience, emotional labor and organizational factors. In the field of teacher job burnout abroad, such research is relatively abundant. For example, the research on the relationship between teacher emotion and job burnout (Chang, 2009), the research on the relationship between teacher self-evaluation and job burnout (Baptiste, 1994), the research on the relationship between teacher teaching style and job burnout (Ghanizadeh and Jahedizadeh, 2016), the relationship between teacher self-esteem or perfectionism and job burnout (Stoeber and Rennert, 2008; Ho, 2016). The mediating role of teacher job burnout, the mediating variable or moderating variable of teacher job stress and job burnout, and the intervention action of teacher job burnout are all the development directions of research on job burnout of EFL teachers in the future.

Thirdly, the research methods need to be improved. At present, the qualitative studies on job burnout of EFL teachers in China mostly adopt the method of structured interview, and the quantitative studies mostly adopt the method of questionnaire, while the qualitative research conducted by foreign scholars since the 1970s and 1980s has adopted a variety of research methods, such as structured interview, semi-structured interview, open interview and clinical observation (Freudenberger, 1975; Maslach and Jackson, 1981; Kalker, 1984). In addition, the countermeasures about job burnout of EFL teachers are mostly based on empiricism, and there are almost no empirical studies on intervention therapy of Chinese EFL teachers’ job burnout and relevant intervention therapy theories. However, foreign scholars have proposed many job burnout intervention therapies, such as Rational Emotive Therapy (Ellis, 1957), Cognitive Behavioral Therapy (Beck, 1976), Mindfulness-Based Cognitive Therapy (Teasdale et al., 1995), Problem Solving Therapy (D’Zurilla and Goldfried, 1971; D’Zurilla and Nezu, 2010), Interpersonal therapy (Donker et al., 2013), Psychodynamic psychotherapy (Johansson et al., 2012), and web-based intervention therapy (Warmerdam et al., 2010), and conducted a large number of empirical studies (Kevin et al., 1983; Pines and Aronson, 1988; Napoli, 2004; Malouff et al., 2007; Andersson and Cuijpers, 2009; Terjesen and Kurasaki, 2009; Richards and Richardson, 2012; Warren and Dowden, 2012; Flook et al., 2013; Andersson et al., 2014; Ozer and Akgun, 2015; Onuigbo et al., 2018; Rosen et al., 2018; Hwang et al., 2019; Fabbro et al., 2020). In view of the shortcomings of intervention research on job burnout of EFL teachers in China, it is necessary to further develop intervention therapy and intervention-related empirical research on job burnout of EFL teachers, so as to alleviate job burnout of EFL teachers.

Fourthly, existing research on job burnout of EFL teachers in China are all cross-sectional or horizontal studies, which can reveal the job burnout of EFL teachers at a certain point or within a short time interval, and belong to the category of static research. However, job burnout is a kind of continuous and negative mental state related to work experienced by individuals (Schaufeli and Enzmann, 1998). The generation of teacher job burnout is a continuous process with dynamic changing characteristics, which may not be perceived by individuals for a long period of time. Therefore, in future, researchers should pay more attention to the longitudinal research on job burnout of EFL teachers in China.

## Conclusion

This review analyzed the recent literature on job burnout of EFL teachers in China. The results indicate that burnout is widespread among the teaching population and has a significant negative impact on teachers’ mental health and work situation. Job burnout of EFL teachers in China is mainly attributed to weak professional development, EFL teachers’ work characteristics, and social expectations and pressure. In addition, demographic variables more or less contribute to the severity of English teacher burnout, among which, middle-aged and elderly teachers, teachers with more than ten years of teaching experience, and teachers with associate titles suffer the most serious job burnout. However, we can also prevent and alleviate the job burnout of English teachers in China at the teacher and school levels respectively, i.e., improving the psychological quality of English teachers and reforming the college management system. In the future, researchers should combine horizontal research with longitudinal research on the basis of existing research results to further expand the breadth, depth and height of the research, so as to provide reliable basis and effective measures for effectively preventing and alleviating job burnout of EFL teachers, improve the career happiness of EFL teachers and improve the effect of EFL teaching.

This review was based on a relatively small size sample analysis, which may not comprehensively reflect the current situation of research on job burnout of EFL teachers in China. This can be regarded as a limitation of this review paper, for a larger-scale analysis of related literature indexed in other databases other than WOS and CSSCI may disclose a clearer and more comprehensive picture of research on job burnout of EFL teachers in China. Another limitation is that this review paper didn’t give definite answers to those questions that researchers hadn’t unanimously reached agreements on, for example, the influence of demographic variables on severity of teacher burnout. However, the aim of the review paper was to analyze the current status of the studies that deal with job burnout of EFL teachers in China, so this limitation hasn’t influenced our work, and it could be a starting point for future research.

## Author contributions

QY designed the study, interpreted the data, and wrote and revised the manuscript. XY revised the manuscript. Both authors contributed to the article and approved the submitted version.
